# Applications of the amniotic membrane in tissue engineering and regeneration: the hundred-year challenge

**DOI:** 10.1186/s13287-021-02684-0

**Published:** 2022-01-10

**Authors:** Hoda Elkhenany, Azza El-Derby, Mohamed Abd Elkodous, Radwa A. Salah, Ahmed Lotfy, Nagwa El-Badri

**Affiliations:** 1 Center of Excellence for Stem Cells and Regenerative Medicine (CESC), Zewail City of Science and Technology, October Gardens, 6th of October City, 12582 Giza Egypt; 2 Department of Surgery, Faculty of Veterinary Medicine, Alexandria University, Alexandria, 22785 Egypt; 3 Biotechnology and Life Sciences Department, Faculty of Postgraduate Studies for Advanced Sciences (PSAS), Beni-Suef University, Beni-Suef, 62511 Egypt

**Keywords:** Natural biomaterial, Amnion, Biodegradability, Regenerative medicine, Tissue engineering

## Abstract

The amniotic membrane (Amnio-M) has various applications in regenerative medicine. It acts as a highly biocompatible natural scaffold and as a source of several types of stem cells and potent growth factors. It also serves as an effective nano-reservoir for drug delivery, thanks to its high entrapment properties. Over the past century, the use of the Amnio-M in the clinic has evolved from a simple sheet for topical applications for skin and corneal repair into more advanced forms, such as micronized dehydrated membrane, amniotic cytokine extract, and solubilized powder injections to regenerate muscles, cartilage, and tendons. This review highlights the development of the Amnio-M over the years and the implication of new and emerging nanotechnology to support expanding its use for tissue engineering and clinical applications.

**Graphical Abstract:**

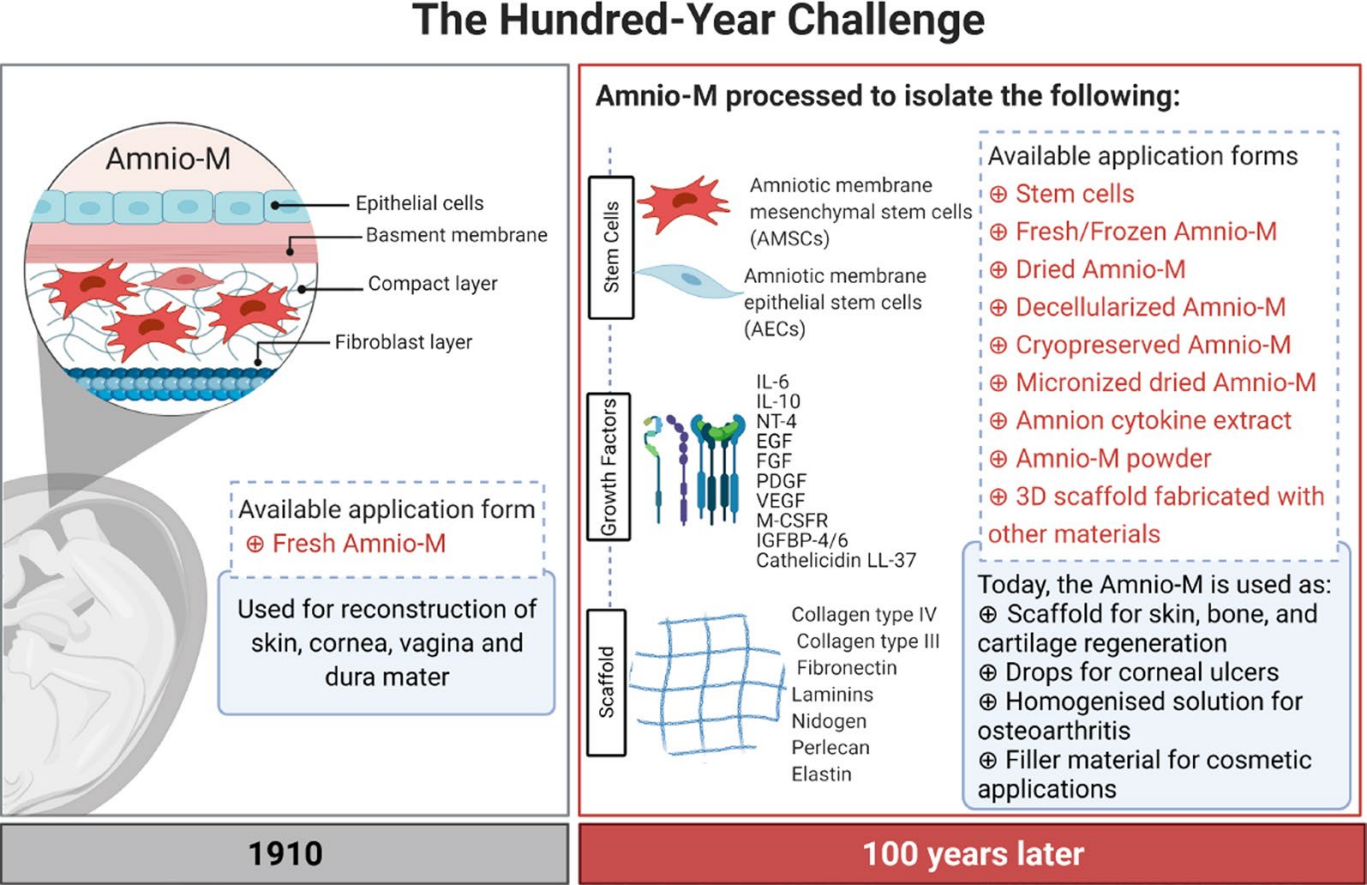

## Introduction

The most inner part of the placenta in direct contact with the fetus is called the amniotic membrane (Amnio-M) [1]. The Amnio-M comprises three layers, an epithelial layer toward the fetus, a basement membrane, and a stroma. The latter consists of a compact layer, a fibroblast layer, and finally, a spongy outer layer [2, 3]. These layers comprise two types of cells: the amniotic epithelial cells (AECs) and the amniotic mesenchymal stromal cells (AMSCs) [4]. Cells of the Amnio-M are essential for regulating the development of the embryo by providing several cytokines and growth factors and contributing to the extracellular matrix (ECM) production [5, 6].

Historically, the applications of the Amnio-M in medical therapy started in the early 1900, when Davis [7] proposed its application in skin transplantation. In 1940, De Rötth [8] proposed its usage as the ideal material to replace damaged conjunctiva instead of the mouth mucous membrane, based on its thin, smooth, and transparent structure that mimics the conjunctiva native tissue. In the same year, Chao, Humphreys [9] successfully used the Amnio-M to reconstruct the dura in experimental severe head injury with dural perforation. The Amnio-M was dried using in an oven or an autoclave and referred to as “amnioplastin,” and used to prevent meningocerebral adhesions and posttraumatic epilepsy [9].

Later, in the early eighties, the Amnio-M was used as an adjuvant to autografts in chronic skin ulcers and to prepare for subsequent skin autograft applications, resulting in successful skin healing [10]. In 1986, the Amnio-M provided an excellent alternative to split skin graft to reconstruct the vagina in vulvovaginoplasty [11]. In an effort to preserve its biological and physical properties, Kim and Tseng [12] proposed cryopreservation of the Amnio-M at − 80 °C. The use of the cryopreserved Amnio-M to reconstruct ulcerated cornea in 11 patients achieved a more than 90% success rate [13]. In 1997, Güler and Ercan [14] were the first to test lyophilized (freeze-dried and sterile) Amnio-M in mandibular vestibuloplasty in which they reported potent angiogenic effect.

To facilitate its application, commercial products of the Amnio-M in the form of suspension are becoming recently available. In 2005, it was first applied in the form of suspension eye drops (AMEED®) for corneal ulcer treatment to overcome the invasive procedure of suturing the Amnio-M graft [15]. In other clinical trials, micronized dehydrated human amnion/chorion membrane (μ-dHACM, EpiFix®) was also shown to be effective in treating diabetic foot ulcers, plantar fasciitis, and osteoarthritis (OA) with minimal invasiveness [16–19]. More recently, the Amnio-M was used as an effective dermal filler for facial wrinkles to restore smooth skin appearance in an in vivo rabbit model [20]. Its cosmetic applications showed rapid improvement in midface aging correction cases, including filling the nasolabial folds, malar fat pad, and descent of lid skin below the orbital rim [21]. The addition of cytokines and growth factors enhanced the usage of the Amnio-M in many other clinical applications in regenerative medicine, such as a three-dimensional (3D) scaffold for tissue engineering and in drug delivery.

The Amnio-M applications have contributed to a better understanding of stem cell biology by providing an optimal platform for cell culture. Our laboratory reported that Amnio-M could provide biologically enriched, well optimized, and topographical mechanical 3D scaffold for culturing stem cells at a low cost compared to the commercially available scaffolds [22]. We also were among the first to provide a microfluidic chip coated with decellularized Amnio-M to introduce a continuous fluid flow to mimic the extracellular fluid dynamics. Recently, the fabrication of Amnio-M organ-on-a-chip has provided an innovative platform for studying the AECs and AMSCs transition and migration in the presence of oxidative stress during preterm birth [23].

**Fig. 1:**
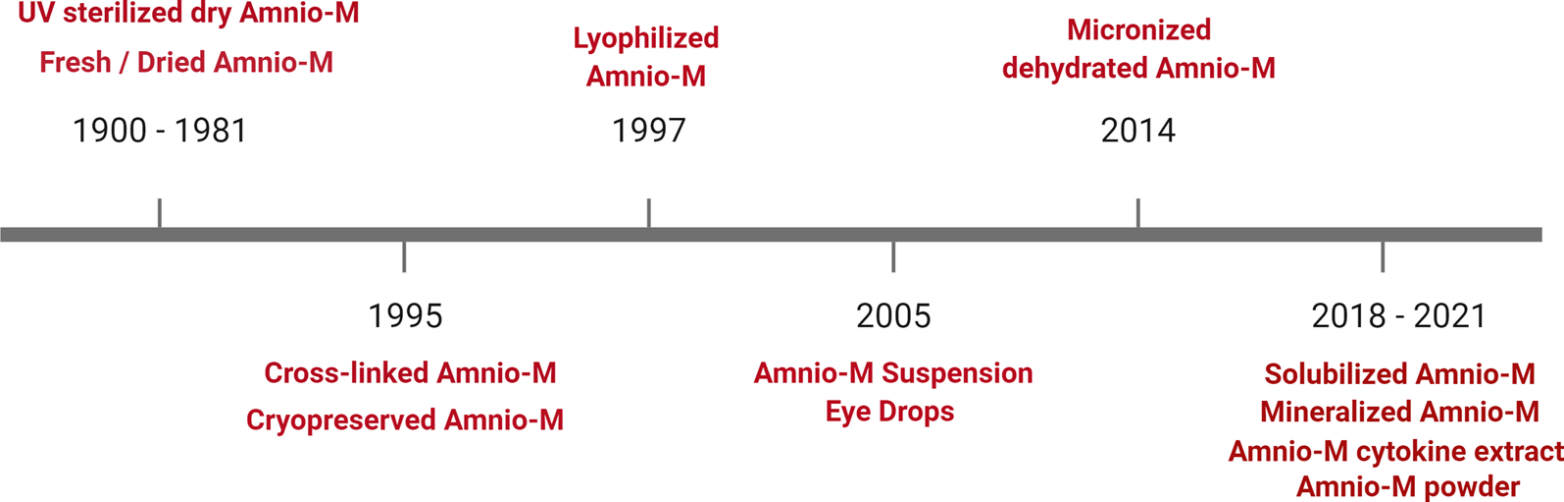
History of Amnio-M modifications and technological enhancement

**Fig. 2:**
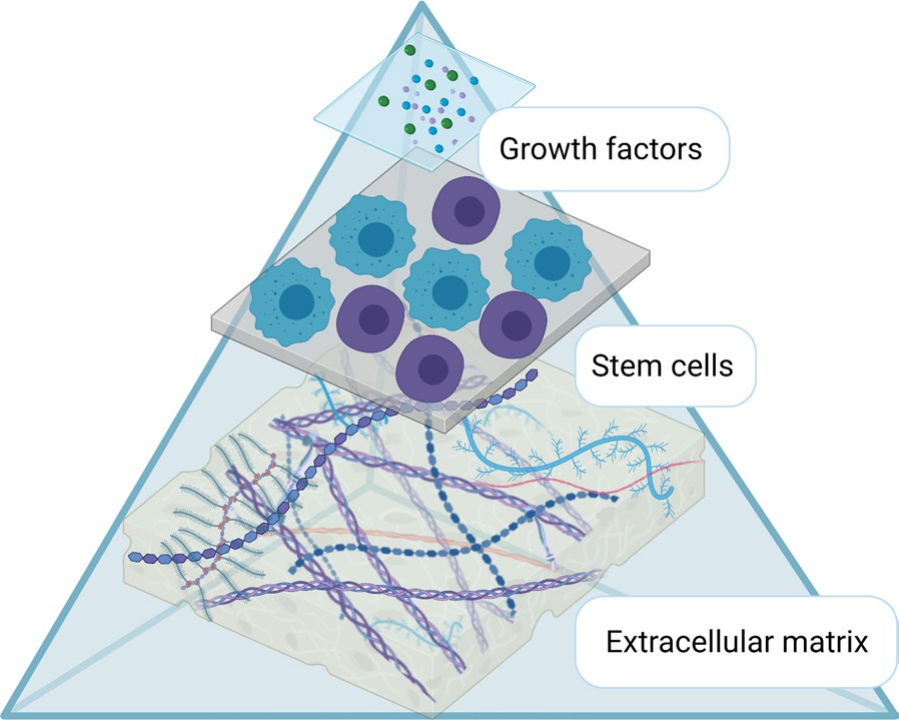
The components of the Amnio-M fulfil the requirements of the “tissue engineering pyramid”

In this review, we highlight the structural and functional properties of the Amnio-M and their role in its diverse applications in regenerative medicine. We also provide an overview of the generation of new forms of the Amnio-M using nanotechnology and their potential applications in tissue engineering. The history of the development of the Amnio-M for research and clinical applications over the past century is summarized in Fig. 1. Figure 2. depicts the main three main components of the Amnio-M and their adaptation to the main pillars of the tissue engineering pyramid that include cells, scaffolds, and growth factors. We will discuss each of these components throughout the review.

## Cellular components of the Amnio-M

The human Amnio-M (h-Amio-M) was shown to include two main types of stem cells, the AECs that rest on a thicker basement membrane and the AMSCs that exist in the deeper spongy layer of the membrane [24, 25]. During embryogenesis, both types of cells originate before the delineation of the three primary germ layers in the pre-gastrulation stages and are typical of epithelial origin [26]. The AMSCs are derived from the extraembryonic mesoderm of the primitive streak, while the AECs are derived from the fetal ectoderm on the eighth day of fertilization and preceding organogenesis [27]. The AECs are specialized fetal epithelial cells that live for less than ten months. The AMSCs are similar to adult stem cells in their capability to differentiate into more specialized cells, such as osteocytes, chondrocytes, adipocytes, cardiomyocytes, myocytes, neurocytes, hepatocytes, and vascular endothelial cells [28, 29]. Unlike embryonic stem cells (ESCs), the AMSCs do not form teratomas upon transplantation in vivo, supporting their safe application for clinical transplantation [30–34]. Other types of stem cells are present in the amniotic fluid (AF), and are known to shed during fetal development from both embryonic and extraembryonic tissues [35]. The AF is formed in the amniotic cavity of early gestation two weeks after fertilization [36]. The first progenitor cells derived from the AF were reported in 1993 [37]. The amniotic fluid stem cells (AFCs) include human amniotic fluid epithelial cells (AF-AECs) and amniotic fluid MSCs (AF-MSCs). The former were reported to have the ability to differentiate into neurons, astrocytes, oligodendrocytes and can be used for transplantation therapy in neurodegenerative diseases [38]. The AF-MSCs express the pluripotent marker Oct-4 and have multiple differentiation capacities similar to the AM-MSCs [32, 35]. They can be easily isolated from the AF and have been used in several therapeutic applications [36, 39]. They were also reported to have no tumorigenicity, low immunogenicity, and minimal ethical concerns [36].

The amnio-M cells are also easily accessible, provide high yield, and represent an ethically acceptable source of stem cells for applications in regenerative medicine. Moreover, cells obtained from the hAmnio-M have high plasticity and show multilineage differentiation potential while presenting no risk of tumorigenicity following transplantation [40]. These advantages render the hAmnio-M desirable use in clinical application, including cardiac repair, neurological reconstruction, bone remodeling, and hepatic regeneration [41]. Of particular interest, the lack of histocompatibility antigens on the surface of the Amnio-M cells supports their applications as an excellent biocompatible scaffold that evades the body’s immune reactions upon transplantation [42].

### Development of the Amnio-M: epithelial-mesenchymal transition

Epithelial-mesenchymal transition (EMT) is a process in which epithelial cells lose their polarity and acquire mesenchymal phenotype. EMT is not a simple binary decision between the mesenchymal and epithelial phenotype but a chain of forwarding and backward dynamic transitional states between the mesenchymal and epithelial fate [43]. EMT is involved in embryological development, wound healing, and stem cell differentiation. In pathological conditions, it plays a role in tumor generation, progression, metastasis, and organ fibrosis [43, 44].

EMT is regulated by a myriad of transcription factors, including tumor necrosis factor alpha (TNF-$\alpha )$, Interleukin 6 (IL-6), IL-8, Zinc finger protein of Snail1 (SNAIL1), SNAIL2, prostaglandin, and extracellular signals such as Wnt, transforming growth factor beta (TGF-β), and fibroblast growth factor (FGF) [45, 46]. Zinc finger E-box (ZEB) and Twist are other crucial EMT regulators that modulate epithelial marker loss, such as E-Cadherin and IL-8, along with up-regulation of mesenchymal markers including vimentin, N-Cadherin, and fibronectin [45, 47].

**Fig. 3:**
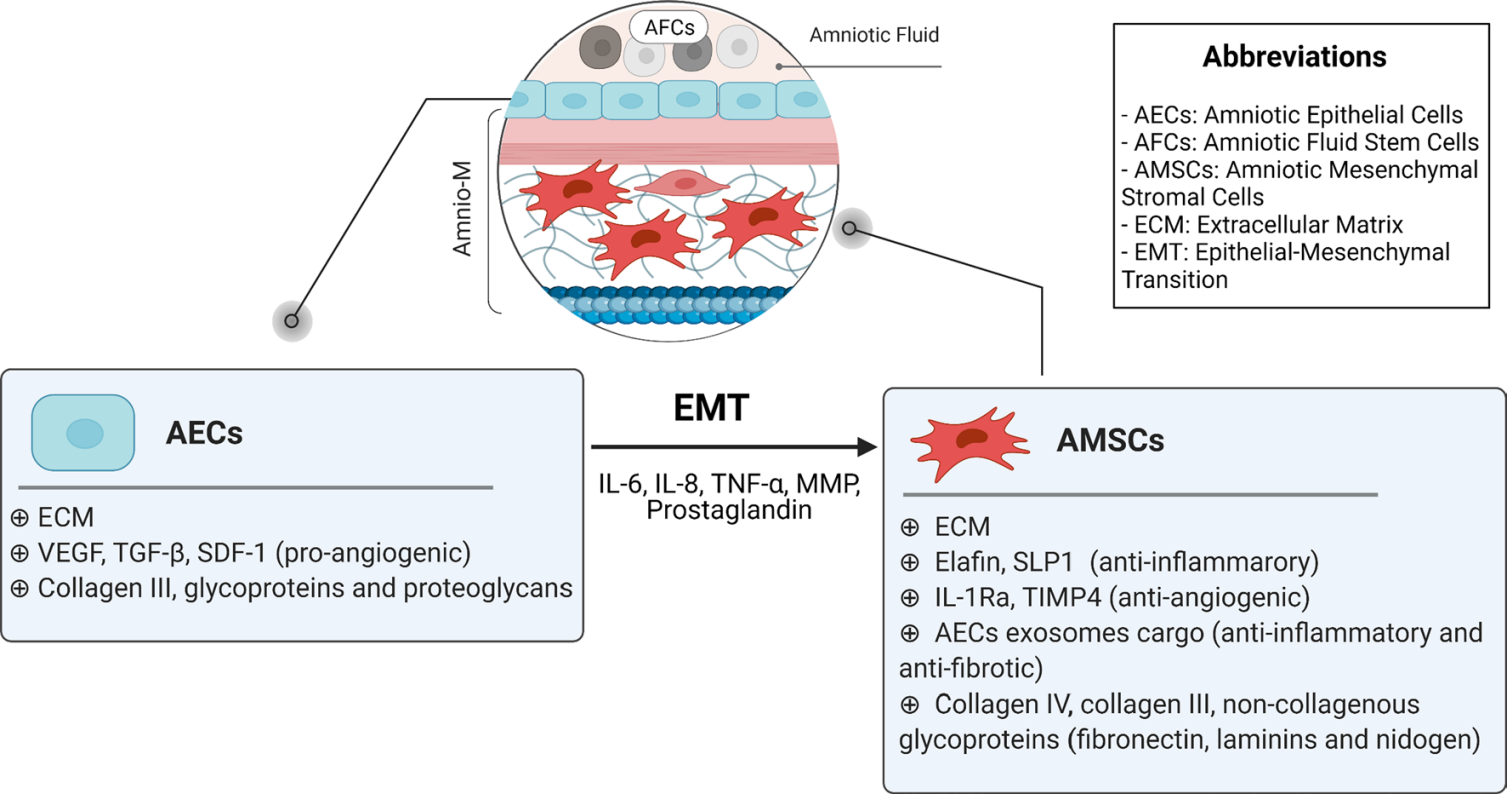
The secretome of the AECs and AMSCs, and the factors controlling EMT between the two cell types. *Abbreviations* Epithelial-mesenchymal transition (EMT); amniotic epithelial stem cells (AECs); amniotic mesenchymal stromal cells (AMSCs)

During in vitro amplification, AECs undergo various cellular modifications toward the AMSC phenotype via spontaneous EMT. As reported by Canciello et al., prostaglandin altered this process by inhibiting EMT, suggesting a significant contribution of EMT in the AEC and AMSC transdifferentiation [45]. EMT-associated cellular events include ECM degradation, cytoskeleton disorganization in addition to alteration in the expression level of intracellular adhesion molecules, enabling the cells to acquire migratory behavior [43, 48]. Although these events were studied and reported in cancerous transformations, the same cellular events were also reported to be associated with the ruptured amniotic membrane [48]. Janzen et al. reported that TNF-α, IL6, IL8, prostaglandin, and Matrix metalloproteinase (MMP) are all EMT inducers and biologically active and in substantial concentrations in the fetal-placental unit preterm. They concluded that EMT plays a role in the amniotic membrane rupture via decreasing its tensile strength just before labor (Fig. 3) [48–50].

### Applications of the AMSCs in regenerative medicine

The rich structure of the Amnio-M supported its use as a natural bio-scaffold for clinical applications. The AMSCs possess unique characteristics that render them useful in diverse applications for tissue repair. These include using as cartilage grafts for tracheal reconstruction of the fetus [51, 52], restoration for the diaphragm muscles [53, 54], bone grafts [55, 56], and heart valve leaflets [57–59]. Furthermore, seeding human AMSCs in gelatin microcarriers could successfully generate modular bone-like tissues upon osteogenic differentiation [60].

In mice, the Amnio-M cells were shown to be effective in treating acute tendinopathy [61], and skin repair [59]. They promoted protection against cellular damage in a liver cirrhosis animal model [62, 63] and improved the heart’s function in a cardiac infarction model [64–67]. Both the AECs and the AMSCs showed promising results when transplanted in diabetic mouse model and effectively brought back glucose to its normal levels [68–70]. This promising therapeutic effect in treating type 1 diabetes has been attributed to the cells’ capacity to differentiate into β-cell in vivo. Furthermore, the AECs have been proposed for spinal cord regeneration, as they expressed neural and glial markers [71] and secreted catecholamine neurotransmitters [72]. For example, injection of AECs in combination with umbilical cord MSCs (UC-MSCs) in spinal cord injury showed significant suppression of microglia activity and reduced neuropathic pain [73].

The AFCs on the other hand were used as an effective cell-based therapy for acute or chronic renal failures and acute tubular necrosis in animal models [74]. The AFCs were reported to facilitate neuroprotection during intercellular coupling due to their high expression levels of gap junction protein [75]. Moreover, the AFCs were found to support intercellular communication with astrocytes, highlighting their role in delivering therapeutic factors, such as microRNAs, to damaged tissues [75].

The regenerative utility of stem cells is not mediated only by direct effects but also via paracrine mechanisms, as shown in animal models [76–78]. Both the amniotic fluid conditioned media (AF-CM) [79] and AMSCs conditioned media (AMSCs-CM) [80] restored blood flow in a murine hindlimb ischemia model. This effect was attributed to the cytokines and pro-angiogenic growth factors released by the cells into the culture medium, including vascular endothelial growth factor (VEGF), TGF-β, and stromal cell-derived factor-1 (SDF-1). AFCs-CM were shown to stimulate endogenous repair mechanisms, such as dermal fibroblast proliferation at the site of injury in a mouse skin wound model [81]. Recruitment of endothelial progenitor cells to ischemic skin in rat models supported therapeutic angiogenesis by delivering angiogenic growth factors and cytokines [82]. In these studies, the potential of both the Amnio-M-derived cells and the AFCs to stimulate tissue repair was mediated by several paracrine mechanisms, such as the release of trophic factors [83], immunomodulation [84, 85], and the establishment of a supportive environment for renewal [86]. Furthermore, both in vitro and in vivo studies showed that the derivatives and protein extracts of the AMSCs and hAECs display potent anti-tumor effects [87–89].

## Amnio-M-derived growth factors and cytokines

**Fig. 4:**
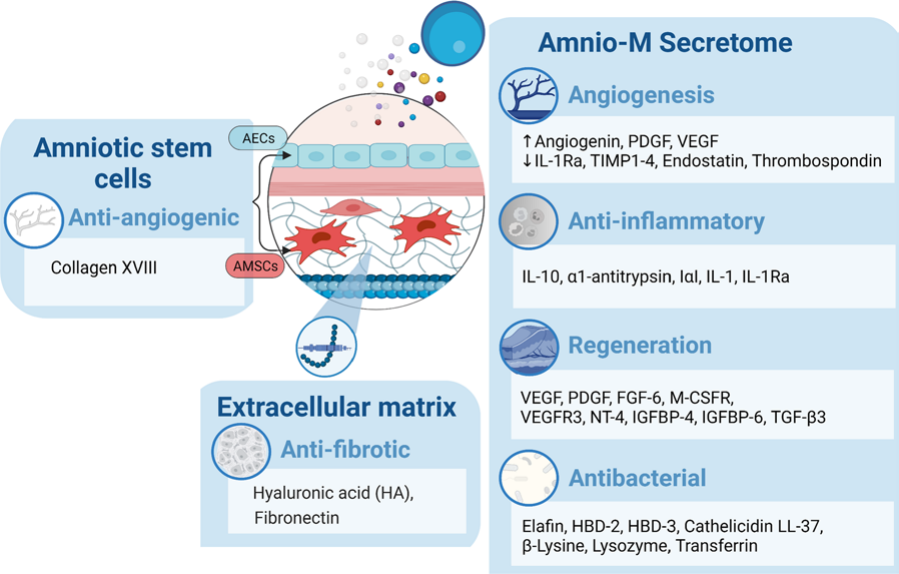
The Amnio-M-derived growth factors and cytokines contribute to wound healing and tissue regeneration by enhancing angiogenesis, reducing inflammation, preventing infection, and reducing scar formation

The anti-inflammatory and antibacterial properties of the Amnio-M are mediated, for the most part, by released growth factors and cytokines. For instance, the angiogenic properties of the Amnio-M were attributed to its capacity to produce VEGF and platelet-derived growth factor (PDGF), both of which mediate wound healing. Moreover, the potent anti-inflammatory and immune-modulatory effects were attributed to the secretion of IL-10 and IL-6 [2, 90]. Hyaluronic acid (HA) in the Amnio-M matrix was reported to inhibit the potent pro-fibrogenic cytokine TGF-β; this could be modulated via increased receptor turnover and decreased endosomal internalization. HA was found to attenuate both SMAD- and non-SMAD-dependent TGF-β1 signaling events [91]. Moreover, Zofia et al. reported that the Amnio-M's secretome contains a wide range of factors that contribute to the regenerative potential and the induction of HUVEC cell migration. These include FGF-6, PDGF-AB, macrophage colony-stimulating factor receptor (M-CSFR), VEGFR3, neurotrophin-4 (NT-4), insulin-like growth factor binding protein 4 (IGFBP-4), and IGFBP-6 [6]. The contribution of the Amnio-M secretome and cytokines in regeneration is summarized in Fig. 4 and Table 1.

**Table 1:** Summary of the relations between the different Amnio-M derived cytokines and their biological functions

Factor	Biological function	Source	Ref
Vascular endothelial growth factor (VEGF)	Wound healing	Amnio-M	[2, 90]
Platelet-derived growth factor (PDGF)	Pro-angiogenic activity		[5]
$\alpha$ 1 anti-trypsin	Protease inhibitors suppress the IL-1-mediated inflammation		[93]
Inter-$\alpha$ -trypsin inhibitor			
IL-1 inhibitors (IL-1RA)			
Fibroblast growth factor 6 (FGF-6)	Increase regeneration potential, Induce HUVEC cell migration	Amnio-M secretome	[6]
Platelet-derived growth factor AB (PDGF-AB)			
Macrophage colony-stimulating factor receptor (M-CSFR)			
Vascular endothelial growth factor receptor 3 (VEGFR3)			
Neurotrophin-4 (NT-4)			
Insulin-like growth factor-binding protein 4 (IGFBP-4)			
Insulin-like growth factor-binding protein 6 (IGFBP-6)			
Elafin	Antibacterial effect		[109]
Secretory leukocyte protease inhibitor (SLPI)	Anti-inflammatory		[6, 92]
Interleukin 10 (IL-10)			
Endostatin	Anti-angiogenic		[103]
Tissue inhibitors of metalloproteases (TIMP-1, 2, 3, 4)			
Thrombospondin -1			
Human β-Defensin 2, 3	Antibacterial effect		[109]
Cathelicidic LL-37			
Collagen XVIII	Anti-angiogenic	AMCs and AECs	[102]
Hyaluronic acid (HA)	Anti-fibrotic	Amnio-M matrix	[91]
Fibronectin	Activation of the ERK pathway		[105]
AECs-derived exosomes	Anti-fibrotic	AECs secretome	[96]

### Immunomodulatory and anti-inflammatory properties

The Amnio-M plays an essential role in combating inflammation via its potential to suppress the pro-inflammatory cytokines. Secreted elafin (peptidase inhibitor 3) and secretory leukocyte proteinase inhibitors were shown to have an anti-inflammatory effect [6, 92], so was IL-10, which is known to suppress the pro-inflammatory cytokines IL-6 and TNF $\alpha$. In addition, the Amnio-M was reported to contain various protease inhibitors that play an essential role as anti-inflammatory mediators such as $\alpha$ 1 anti-trypsin, inter-$\alpha$ -trypsin inhibitor, and IL-1 inhibitors (IL-1RA) that suppress the IL-1-mediated inflammation [93]. Interestingly, the anti-inflammatory action of the Amnio-M was attributed to its ability to trap the inflammatory cells which undergo apoptosis, making it an excellent candidate for transplantation on the ocular surface [94].

Exosomes are nano-sized extracellular vesicles that contain a wide range of bioactive molecules such as nucleic acids, lipids, and proteins. These vesicles participate in intercellular communication and regulate various intracellular biological functions [95]. Tan et al. reported that AECs-derived exosomes mediate an anti-inflammatory response by augmenting macrophages’ phagocytosis properties along with diminished neutrophil myeloperoxidases and inhibition of T cell proliferation. The same group also reported that administering specific doses of AECs-derived exosomes along with bleomycin, an anti-cancer drug, reduced lung inflammation and fibrosis, in addition to increasing the bronchoalveolar stem cell proliferation [96]. The anti-inflammatory effect of the AEC's exosomes was attributed to their effect on decreasing neutrophil myeloperoxidase (MPO) activity, increasing the phagocytic activity of macrophages, shifting their polarization state toward M2, and repressing proliferation activity of CD3 and CD28-activated T cells [5]. Several studies showed that AECs transplanted into immunocompetent animals were highly tolerated and survived in host tissues. These studies confirmed the clinically relevant value of the low immunogenicity displayed by the Amnio-M. This low immunogenicity was attributed to low expression of HLA IA, besides negative expression of CD86, CD40, CD80, and HLA-DR immunogenic markers [97–99]. Furthermore, AEC and AMSCs failed to induce T-cell proliferation in mixed lymphocyte reactions, further confirming their low immunogenicity in vitro [2].

Kupo et al. investigated the immune response generated by xenotransplanted human Amnion-M in the limbus, intracorneal space, and under the kidney capsule of immunocompetent rats. In these experiments, all intracorneal transplanted grafts were tolerated, as well as the grafts under the kidney capsule. However, the latter did not show much host-integrated vascularization when compared to skin graft controls. Interestingly, the skin graft controls showed signs of immune rejection, confirming the superiority of the amnion-M in overcoming graft rejection [100]. In another study by Cargnoni et al., infusion of placenta-derived cells in a lung fibrosis animal model significantly reduced the numbers of infiltrated neutrophil and fibrosis severity [101].

### Dual-effect on angiogenesis

The Amnio-M produces several potent anti-angiogenic factors, including endostatin, tissue inhibitors of metalloproteases (TIMP-1, 2, 3, and 4), and thrombospondin -1 [6, 92]. Both the AMSCs and AECs have been shown to express Collagen XVIII, which displays anti-angiogenic properties [102]. AECs, in particular, were reported to secrete IL-1Ra, TIMP4, and 3, which are known for their anti-angiogenic activity in addition to their anti-cancer properties [103]. AECs were able to suppress capillary formation, as evidenced by aortic ring assay in vitro [104]. Interestingly, pro-angiogenic activity was also reported in the Amnio-M and was found to differ from one cell type to another. This could be attributed to the angiogenesis inducers such as angiogenin, PDGF, and VEGF secreted by the AMSCs, proposing them a candidate for skin ulcer treatment and wound healing [5]. In addition to the cellular component, both the integrin and fibronectin protein content in the ECM of Amnio-M have been demonstrated to interact with PDGF, EGF, and b-FGF growth factors for activation of the ERK pathway [105]. A recent study by Tsai et al. demonstrated that the Amnio-M could be considered an excellent matrix for establishing mature vascular constructs. This is due to its potential for enhancing integrin expression, platelet-endothelial cell adhesion molecule-1, and adhesion molecules such as VE-cadherin in the cultured endothelial cells [106].

### Anti-fibrotic effect

The Amnio-M showed an anti-fibrotic effect via the downregulation of the expression of TGF-β3 and its receptor, promoting wound healing instead of scar formation. TGF-β3 is an antagonist for TGF- β1 and TGF-β2, which stimulates ECM synthesis, increases collagen deposition in the wound area and promotes scar formation [107]. Tseng et al. reported that the Amnio-M stromal matrix could exert direct and potent anti-scarring action on the ocular surface fibroblasts via suppressing TGF-β transcription and signaling [93]. AEC-derived exosomes showed anti-fibrotic properties by virtue of their protein cargo involved in EFG, FGF, and PDGF signaling pathways [96]. In addition, anti-fibrotic miRNAs were found in AEC’s exosomes that target various aspects of TGFβ signaling [96].

### Antibacterial properties

The antibacterial properties of the Amnio-M was shown against both gram-positive and gram-negative bacteria. Zare-Bidaki et al. reported the significant growth inhibitory effect of both the amniotic and the chorionic membranes against eight bacterial strains using disk diffusion assays. These included *Escherichia coli, Bacillus cereus*, *Klebsiella pneumonia*, *Streptococcus pyogenes*, *Pseudomonas aeruginosa*, *Staphylococcus aureus*, *Shigella flexneri* and probiotic *Lactobacillus plantarum* [108]. In the same direction, Tehrani et al. tested the Amnio-M extract before and after its exposure to IL-1β against *Pseudomonas aeruginosa* and *Staphylococcus aureus*, in addition to two clinically isolated sensitive strains of *Escherichia coli*. The data showed that pre-exposure of the Amnio-M to IL-1β augmented the antibacterial peptide secretion, including elafin, HBD-2, HBD-3, and cathelicidic LL-37, which in turn enhanced the antibacterial properties of the membrane [109].

A clinical study that compared the therapeutic effect of autologous skin graft and Amnio-M dressing in 33 patients suffering from burn showed that the latter was more effective in alleviating the pain, fastening the healing and epithelialization, and protecting the wounds from infection [110]. Moreover, anti-microbial agents in the AF such as beta-lysin, bactericidin, lysozyme, and transferrin could be involved in mounting that effect [92]. The antibacterial potential of the Amnio-M may also be attributed to its sealing capacity. After implantation, the Amnio-M lies in direct and very close contact with the underneath layers and form a firm adherent shield with the wounds, preventing any contamination and enabling lymphatic integrity at this site, as hypothesized by Copra et al. [111].

## Extracellular matrix (ECM) component of Amnio-M

The 2D monolayer cell growth lacks faithful mimicry of the biological tissue complexity [112]. 3D natural scaffolds, such as the Amnio-M, or synthetic scaffolds, such as polymer-based scaffolds, play a critical role in supporting cell growth, proliferation, and differentiation [113]. The Amnio-M ECM comprises a cross-linked network of dynamic macromolecules, provides structural support, and acts as a physical scaffold for cells in various body tissues [114]. The Amnio-M possesses unique biophysical and biochemical characteristics that modulate various cell functions such as wound healing and vascularization [115, 116]. In addition, it organizes cells in the space of tissues, controls cell regulation by environmental signals, and activates intracellular signaling by binding with specific transmembrane receptors [117, 118].

### Chemical composition of the ECM

Cell attachment to a specific scaffold is controlled by various components of the ECM [119]. The absence of specific ECM molecules, such as laminin, fibronectin, and collagen within the scaffold's basement membrane, has a significant impact on cell growth and adhesion [120]. The ECM’s multiple components act as adhesion and signaling ligands and have a significant role in cell proliferation, migration, and differentiation [116].

The Amnio-M comprises three main layers: an epithelial monolayer, a thick basement membrane, and an avascular stroma [121]. The AECs secrete collagen types I, III, IV, V, VII and non-collagenous glycoproteins, including fibronectin, laminin, and nidogen, all of which constitute the basement membrane of the Amnio-M [119, 122]. On the other hand, a non-fibrillar network of type III collagen, hydrated glycoproteins, and proteoglycans is commonly found in the spongy layer of the stromal part of the amnion [123, 124]. Non-sulfated glycosaminoglycans, such as HA, multiple types of cytokines, proteases, and protease inhibitors, are all significant factors in wound healing [125]. Furthermore, Amnio-M was reported to contain an abundant number of heavy chains of inter-α-inhibitor (HC·HA) combined with human pentraxin 3 (PTX3, TNF-inducible gene 14 protein) [126, 127]. Additionally, perlecan, a large heparan sulfate proteoglycan, is a crucial component of the basement membrane [128, 129]. Perlecan has an essential role in growth factor binding and interactions with many extracellular proteins and molecules responsible for cell adhesion [130].

### Mechanical properties of the ECM of the Amnio-M

The mechanical properties of the Amnio-M, such as elasticity, stiffness, and other biomechanical characteristics, are attributed to its ECM, which depends on the variation in its components, including proteoglycan, elastin, and collagen [131]. The Amnio-M exhibits a time-dependent mechanical response and viscoelastic properties [132]. These mechanical properties vary depending on the stage of the Amnio-M. For example, the preterm (26–36 weeks) Amnio-M was found to possess higher mechanical integrity compared to full term Amnio-M (36–40 weeks). However, the stiffness of the term Amnio-M was more adaptable for most tissue engineering applications [119].

The utility of the of the Amnio-M in tissue engineering is highly dependent on its elastic characteristics. Elasticity is defined as the material’s ability to withstand a distorting force and to return to its original shape and size after that force is removed. It is characterized by Young’s modulus, which is the ratio of applied stress to strain and measured in Pascals (= N/m^2^) and can be found using the following formula *E* = *α*/*ε*, where *E* is Young’s modulus, *α* is applied stress, and *ε* is the strain [133]. Young’s modulus of preterm human Amnio-M is reported to be 3.6 × 10^6^ Pascal (3.6 MPa) and about 2.29 × 10^6^ (2.29 MPa) for full-term human Amnio-M [119]. Benson-Martin et al. reported an inverse relationship between the thickness of Amnio-M and the elastic modulus (stiffness of the material). The thicker (proximal, adjacent to placental disk) Amnio-M has lower Young’s modulus (less stiff) compared to the thinner (distal, handbreadth from the placental disk) Amnio-M (more stiff). One possible explanation may be attributed to variance of the alignment of the collagen fibers, which constitute structure bulk of the Amnio-M [134]. Recently, the distal Amnio-M was shown to possess a higher degree of anisotropy (increase the stiffness of fibrillar materials) within its collagen fiber arrangements than the proximal Amnio-M [134]. This change in mechanical properties may be attributed to the content of collagen. It is also worth mentioning that elastin present in the fetal amnion was reported to provide the molecular basis for Amnio-M elasticity [135].

## Essential considerations for biomedical applications of the Amnio-M

### The source of the Amnio-M

**Fig. 5:**
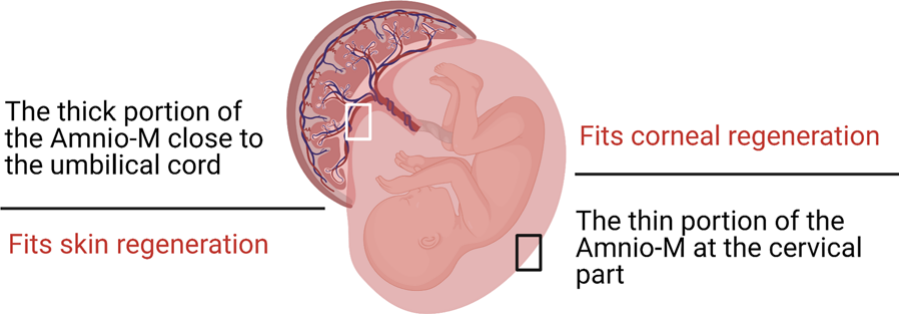
Site selection of the Amnio-M based on its thickness to fit various clinical applications

The source of the Amnio-M source, whether after natural delivery or via Cesarean section, was found to impact its physiology, integrity, growth factors content, and availability. The Amnio-M donor should be screened to avoid transmission of infectious diseases [136], and especially after the COVID-19 pandemic due to fears of vertical transmission [137]. Litwiniuk, Radowicka [138] reported that Cesarean section-derived cervical portion of the Amnio-M stimulated the proliferation of keratinocytes more than that of fibroblasts. These data were valuable in the application of the Amnio-M grafting for ocular defects. Furthermore, natural delivery is associated with decreasing the Amnio-M tensile strength just before labor due to the EMT process. This results in the loss of cytoskeleton organization and intercellular adhesion molecules as described above. The thickness of the membrane also varies from 0.02 to 0.5 mm according to the anatomical site, which may impact its clinical applications [139]. The thickest part lies toward the umbilical cord (placental amnion), while the opposite part is thinner and more transparent (peripheral amnion), which lends itself to superior corneal grafts (Fig. 5) [140].

### Processing, preservation and sterilization of the Amnio-M

Over the past century, the Amnio-M use has benefited from technological advantages, preparation techniques, and sterilization methodologies. At the beginning of the twentieth century, the Amnio-M was used immediately after harvesting during delivery to cover skin lesions [7]. Because of the immunogenic response, and the lack of amnion banks, it became necessary to find other methods for preserving and processing the Amnio-M. In 1938, Chao, Humphreys [9] proposed to dry the Amnio-M before usage to avoid the irritation induced by fresh samples. The membrane was air or oven-dried, then sterilized by either autoclaving or boiling. However, the latter treatment was quickly discounted as it resulted in shrinkage and disruption of the membrane. Drying the Amnio-M was a turning point in its usage in tissue reconstruction, as it proved to be safe, effective and solved the storage deficiency of the fresh membrane. With the advances in sterilization techniques, Rao and Chandrasekharam [141] used ultraviolet (UV) sterilization. Their data showed that irradiation did not affect the biological and physical properties of the Amnio-M. Other methods for sterilization of the Amnio-M include the use of peracetic acid and organic peroxides. These chemical factors were shown to be effective and also safe compared to sterilization by irradiation, with minimum effect on collagen content [142].

In the nineties, Kim and Tseng [12] proposed cryopreservation of the Amnio-M by storing it in − 80 °C using a storage medium composed of glycerol in Dulbecco’s Modified Eagle Medium (DMEM) (1:1). The advantages of cryopreservation were most evident in maintaining the integrity of the ECM. However, glycerol was reported to maintain cell viability, as well as high bFGF production for no more than 3 months of storage [143]. More investigations are needed to find an optimal cryo-preservative that can maintain the Amnio-M biological content and physical properties for more extended periods. In 2004, Nakamura and Yoshitani [144] proposed a new preservation technique to freeze-dry the Amnio-M (FDAM) by incubating the membrane with EDTA for 2 h then freeze-drying it under vacuum at room temperature. This technique was as effective as cryopreservation in effectively retaining the biological, physical, and histological properties of the Amnio-M. Compared to the dried Amnio-M, the fresh-frozen membrane showed negligible differences in the membrane stability, although the content of the epidermal growth factor (EGF) was shown to be higher in the dried membrane [145].

Recent attempts to prepare the Amnio-M in an injectable solution has been promising to reduce its grafting procedure's invasiveness, especially for corneal ulcers and osteoarthritis. This suspension could be marketed either in the form of an amnion cytokine extract (ACE) or amniotic membrane extract eye drops (AMEED). ACE was reported to reduce the clinical symptoms of dry eyes [146]. In contrast, AMEED was reported to efficiently treat dry eyes, chemical ulcers, and diffuse limbal stem cell deficiency (LSCD) [147]. In osteoarthritis, the Amnio-M was a part of μ-dam (EpiFix®) product, which showed promising efficacy in ameliorating the arthritis symptoms [16, 148].

Other forms of the Amnio-M include gel and sponge, both used for cartilage regeneration [149]. Gel formation was performed by collagen extraction from the Amnio-M after 24 h incubation with guanidine solution (4 M) suspended in Tris buffer. The sponge scaffold was fabricated by precipitation collagen type I using acetic acid followed by freezing and drying. The extracted collagen in this study has shown high hydrophilicity, biocompatibility, and induced cartilage formation [149]. Other similar components were extracted from the Amnio-M, such as hyaluronic acid and PTX3, both of which had well-known effect on healing and reducing scar formation. Tseng and colleagues [126] purified HC·HA from the Amnio-M. This active component has shown a crucial role in both reducing scar formation and inflammation, which were attributed to suppression of TGF-β1 and inducing macrophage death. Later, human PTX3 was reported to be integrated with HC·HA to form AM HC-HA-PTX3 and was efficiently extracted from the Amnio-M using agarose overlay [127]. Interestingly, PTX3 has been reported to play a role in polarization of M2 macrophages which is linked to phagocytosis of apoptotic cells [127, 150]. In summary, new advanced technology helped in providing the Amnio-M in different forms, rather than the fresh membrane, as cryopreserved Amnio-M, FDAM, Amnio-M suspension, gel and sponge form (Table 2). Also, several components have been extracted to be used in regenerative medicine as collagen, HC·HA and HC-HA-PTX3.

**Table 2:** Comparison of advantages and disadvantages among the different methods of Amnio-M sterilization and preparation

	Advantages	Disadvantages	Ref
**Sterilization technique**			
Boiling	Cheap and liable method	Shrinkage and disruption of the membrane	[9]
Autoclave	Safe, effective, and low cost		[9]
Peracetic acid	Retaining more Collagen types I and III than gamma radiation		[142]
Irradiation	No effect on the biological and physical properties of the Amnio-M	Lessening of growth factors content	[141, 186]
	Storage for up to 5 years		[187]
**Preparation technique**			
Fresh frozen	Membrane stability	Low EGF content High degradation rate	[145]
Drying	Membrane stability similar to fresh frozen, higher EGF content	Collagen -VII and laminins were not detected compared to cryopreserved	[145, 188]
Cryopreservation	Maintaining the integrity of the ECM high bFGF content	Cell viability and growth factors decreased after 6 months of storage	[143]
Lyophilization	Retained the biological, physical, and histological properties similar to cryopreservation	TGF-β and bFGF levels lower than fresh	[144]
		Due to the irradiation process	[187]
Decellularization + lyophilization	Maintained type IV and type V collagen, elastin and laminin Higher mechanical properties compared to fresh	Thinner membrane compared to fresh	[189]
Amnio-M sponge	3D Scaffold that can fill the tissue gab	TGF-β and bFGF levels lower than lyophilized membrane	[187]
Amnion cytokine extract	Facilitate application as it can be injectable or applied as an eye drop		[146]
Gel form	Collagen with high hydrophilicity, biocompatibility, and induced cartilage formation		[149]

### Enhancement of the Amnio-M biomaterial (3D) properties

There is a complex set of requirements that must be taken into consideration when choosing the suitable scaffold to meet the morphology and functionality of the native tissues. Many attempts were reported to modify the Amnio-M to match the ideal scaffold characteristics regarding degradability, porosity, surface roughness, hydrophilicity, delivering bio-active molecule, biocompatibility, deliverability (easy to deliver), and mechanical reliability [151, 152].

#### Cellularity

To ensure biocompatibility, the decellularization strategy of the Amnio-M evolved to decrease the immunogenic response generated by the in vivo implantation of the membrane. The Amnio-M’s decellularization (removal of the cellular compartment) process was reported to have no adverse effect on intact collagen types I, III, and IV, which will favor biocompatibility [153]. Of note, decellularization results in loss of the stem cell content of the Amnio-M, leading to a lower content of growth factors and cytokines. This encouraged many researchers to use the non-decellularized Amnio-M in preparing Amnio-M extracts or even the Amnio-M powder [154].

#### Biodegradability

##### Cross-linking

The fresh cryopreserved membranes take about seven days to degrade by enzymatic digestion [153]. This fast degradation is considered a serious limitation in its usage for skin regeneration, as skin substitutes should stay at least two weeks to vascularize sufficiently [155]. Importantly, many tissue defects required a long-lasting scaffold until complete recovery. To satisfy these requirements, in 1994, Spira et al. investigated the cross-linking of Amnio-M extracted collagen using radiation. This cross-linking resulted in the detection of almost 70% of the Amnio-M collagen after 12 months when implanted subcutaneously in a rat model [156]. Similarly, in 1999, the Amnio-M was cross-linked with either chemicals or radiation, which not only resulted in a highly stable membrane, (for up to 90 days) but also enhanced the mechanical properties of the membrane without any adverse effects on cell attachment or viability [157]. Furthermore, cross-linking using radiation by γ-ray, UV irradiation, and electron beams was shown to be effective and safe [158, 159]. Later on, a larger set of chemicals was shown to be safe and effective in cross-linking the Amnio-M. For example, aluminum sulfate (Al_2_(SO_4_)_3_) showed a significant increase in its tensile strength [160]. Also, carbodiimide enhanced the physicochemical properties of Amnio-M, enhancing the differentiation potential of human limbal epithelial progenitor cells [161]. A combination of carbodiimide and N‐hydroxy‐succinimide caused effective cross-linking sufficient for promoting optimum mechanical and optical properties for corneal regeneration [162]. Recently, the natural extract genipin has been used to cross-link Amnio-M and enhance biostability and biocompatibility [163]. Table 3. summarizes the cross-linking techniques to control the Amnio-M biodegradation rate.

**Table 3:** Overview of the advanced modalities used to enhance Amnio-M for clinical applications

Enhancement modalities	Additives	Purpose	Membrane status	Study type	Outcome	Ref
Cross-linking	Glutaraldehyde γ-ray and electron beam irradiation	Testing degradation rate	Decellularized Amnio-M	In vitro & in vivo	GA-cross-linked Amnio-Ms were degraded more slowly with a slight tissue response. γ-ray and electron beam irradiation decreased the tensile strength	[157]
	Glutaraldehyde	Corneal regeneration	Intact Amnio-M	In vitro and clinical cases	High mechanical properties in comparison with fresh and cryopreserved membranes. Low degradation rate and better transparency	[158]
	Al2(SO4)3	Corneal regeneration	Intact Amnio-M	In vitro	Al2(SO4)3 increased the tensile strength of the membrane	[160]
	Carbodiimide	Corneal regeneration	Decellularized Amnio-M	In vitro & in vivo	0.05 mmol EDC/mg support cell proliferation and maintained differentiation of LEC	[190]
	Photo cross-linking UV irradiation	Corneal regeneration	Intact Amnio-M	In vitro	Biocompatible membrane, with detectable maintenance of cell stemness	[159]
Hybridization With natural or synthetic materials	Atelocollagen skin collagen	Skin regeneration	Bovine decellularized Amnio-M	In vivo/ pig model	Inhibit inflammatory reactions and promote wound healing	[191]
	Hyaluronic acid hydrogel	Skin regeneration	Human solubilized Amnio-M	In vitro & in vivo	In vitro, the proposed scaffold enhanced cell proliferation. In vivo, it enhanced wound healing, reepithelization, and vascularization	[171]
	GelMA hydrogel	Oral mucosa regeneration	Decellularized Amnio-M particles	In vitro & in vivo	GelMA–dAmnio-M Particles scaffold has been proven to be effective in neovascularization and mucosa repair	[172]
	Aloe vera gel	Skin regeneration (burn)	Non-decellularized membrane (powder)	In vitro and in vivo	Significantly enhance burn wound healing	[175]
	Nano-fibrous Fibroin	Skin regeneration	Decellularized hAmnio-M	In vitro	Bilayer Amnio-M/nano-fibrous fibroin scaffold represents an efficient natural construct with broad applicability to generate keratinocytes from Menstrual stem cells	[174]
	POC polymer	Cleft palate repair	Decellularized hAmnio-M	In vitro & in vivo	The biocompatible scaffold could regenerate both soft and hard tissue effectively	[192]
Combination with cells	Dental pulp derived cells	Periodontal tissue regeneration	Decellularized hAmnio-M	In vitro	cell sheet that contained MSC may be helpful for application in periodontal tissue regeneration	[182]
	TGF‐β3 BMSCs	Skin regeneration	dehydrated Amnio-M (hDAM) commercial	In vitro & in vivo	Wound healing with a minimal scar in a full-thickness wound in rat back	[183]
	Corneal stromal cells (CSCs)	Cornea regeneration	ultrathin Amnio-M	In vitro and in vivo	UAM provided a suitable scaffold for CSCs to generate tissue mimic the native cornea	[193]
	ASCs	Skin regeneration	Decellularized hAmnio-M	In vitro and in vivo	AM-ASCs accelerated the wound healing with a less inflammatory response in a third-degree burns rat model	[184]
Drug carrier Nanoreservoir	Cefazolin	Cornea regeneration	hAmnio-M	In vitro	High drug entrapment was achieved by incubation of Amnio-M for 3 h at 4C	[179]
	Moxifloxacin	Cornea regeneration	hAmnio-M	In vitro	Thick HAM entraps moxifloxacin efficiently higher than thin HAM. 3 h incubation was sufficient for entrapment	[180]
Other additives	Tissue glue	Cornea regeneration	Intact Amnio-M	Clinical trial (After dermoid removal)	Rapid corneal reepithelization and smooth healing	[194]
	Amino acids	Cornea regeneration	Carbodiimide cross-linked Amnio-M	In vitro and in vivo	Lysine amino acid could increase the cross-linking efficiency of Amnio-M	[195]
	Calcium and Phosphate	Bone regeneration	Decellularized hAmnio-M	In vitro and in vivo	The mineralized Amnio-M enhanced ASCs osteogenic differentiation in vitro and bone regeneration in a calvarial bone defect in vivo	[181]

##### Integration with other material

3D scaffold production was developed to overcome the biodegradability issue by means of fabrication of Amnio-M. In 2011, a preliminary study was conducted to produce a fabricated Amnio-M-fibrin scaffold that effectively enhanced chondrogenic differentiation with a slow degradation rate [164]. Nanofibrous silk fibroin was also tested to produce a 3D bioscaffold, which was shown to extend the degradation time to two weeks [131]. Microfabricated electrospun membranes composed of poly-lactic co-glycolic acid (PLGA) and Amnio-M in equal amounts showed a delay in the degradation rate, pushing it to completely degrade after four weeks [165]. Integration with other material was also indicated to overcome the weak mechanical properties of Amnio-M. Adamowicz, Pokrywczyńska [166] proposed covering the Amnio-M with electrospun poly-L-lactide-co-E-caprolactone (PLCL) from both sides to be grafted in the wall of urinary bladder. The degradation of this biomaterial in vivo ranged between 8 and 10 weeks. Also, higher elasticity similar to normal biomechanical properties of the urinary bladder wall was reported.

#### Orchestrating tissue healing and regeneration

Biomaterials used in tissue engineering for clinical applications should display desirable effects such as suppressing inflammation, reducing scar formation, enhancing vascularization, preventing infection, and recruiting native resident stem cells to the site of injury. The content of proteins, cytokines and growth factors in the Amnio-M propose its use as an optimal biomaterial for wound healing. Using the Amnio-M as a single or even double sheet has a wide range of applications for covering injuries at the outer exposed surfaces of the body, such as the skin and the cornea. Moreover, the Amnio-M can also act as a protective barrier for internal organs. For example, wrapping of peripheral nerves, the spinal cord, the peritoneal cavity, and tendon with the Amnio-M could prevent adhesion and reduce scar formation [167–170].

Importantly, the regenerative potential of the Amnio-M is not limited to simple usage as a coverage bandage but extends to include its high content of regenerative key factors. Advanced technologies supported the transformation of the Amnio-M into 3D scaffold that can fit appropriately into a defect. They have also helped its reintegration with other natural and synthetic biomaterials as shown in Table 3. This integration could allow for superior applications. For example, the gel form of the Amnio-M would be advantageous over powder or cryopreserved Amnio-M in wound healing as it will provide a hydrating wound barrier, overcome the wound contractility, and control the rate of release of therapeutic components [154].

In a recent study, human solubilized Amnio-M (prepared by lyophilization) was combined with hyaluronic acid and cross-linked to evaluate its application in skin wound regeneration [171]. Accelerated healing and reepithelization, as well as noticeable neovascularization, were achieved. Similarly, combination of Amnio-M powder with methacrylated gelatin (GelMA) hydrogel could enhance mucosa regeneration through enhancing vascularization [172]. The same group used GelMA-Amnio-M composite in skin regeneration, showing adequate collagen deposition, as well as proper mechanical properties [173]. Integration of the dAmnio-M with nano-fibrous fibroin enhanced the in vitro differentiation of menstrual blood MSCs into keratinocyte [174]. Furthermore, Amnio-M powder mixed with Aloe vera gel enhanced in vitro proliferation, migration and adhesion of fibroblast and keratinocytes. In vivo studies showed that the gel did not cause irritation and accelerated the wound healing with minimal scar formation. However, precautions should be taken for high Aloe vera concentration as it showed cytotoxic effects in vitro [175].

One of the most exciting developments of the Amnio-M is its optimization in producing sutureless membranes to facilitate its applications, especially in the corneal defects. Fibrin glue has been proposed by Szurman, Warga [176] as a bio-adhesive to stabilize the Amnio-M over the corneal surface. However, in some cases, such as Stevens-Johnson syndrome (SJS) and toxic epidermal necrolysis (TEN) which require covering the entire cornea, the conjunctiva, as well as eyelid, securing a large sheet of Amnio-M was challenging. Shanbhag, Chodosh [177] proposed cyanoacrylate glue to fix Amnio-M into the eyelid skin alongside using a silicon ring to stabilize it over the cornea. Another study on the treatment of recurrent retinal detachment using Amnio-M has shown that adding platelet-rich plasma (PRP) increased the success rate of sealing the retinal hole [178].

Recently, the drug reservoir properties of the Amnio-M have been investigated. They were shown to effectively deliver bioactive molecules such as cefazolin and moxifloxacin, where the Amnio-M could sustain their release for up to 7 weeks [179, 180]. Furthermore, the Amnio-M was loaded with calcium and phosphate using the double diffusion method to develop a mineralized membrane capable of bone regeneration [181]. It is worth mentioning that Amnio-M was investigated for effectively acting as a carrier for stem cells delivery from different sources (Table 3). These include the bone marrow, adipose tissue, dental pulp, and menstrual blood [174, 182–185]. Decellularized Amnio-M provided a biocompatible ECM for culturing DP-derived cells and retaining their properties and provided cell sheet that favors its application in periodontal tissue regeneration [182]. The dAmnio-M loaded ASCs have shown potent anti-inflammatory effects and fastened skin wound healing in burn animal models [184]. Similarly, dehydrated Amnio-M loaded with genetically modified TGF-β3 BMSCs significantly reduced scar formation and improved the cosmetic appearance in full-thickness wounds [183].

## Conclusions

According to the tissue engineering pyramid, successful tissue engineering and regeneration can be accomplished by integrating several factors including scaffolds, cells, vascularization, growth factors, and chemical and physical cues. The Amnio-M cover most of the tissue engineering pyramid component as it can provide appropriate ECM, cells and different kinds of growth factors [152]. This wide range of cover in tissue engineering encouraged researchers to develop the membrane using advanced technologies to modify and enhance these unique and valuable properties. These modifications aimed to increase biocompatibility by decellularizing the membrane and facilitating the deliverability through producing Amnio-M suspension as AMEED and μ-dHACM that can be injected rather than sutured. Furthermore, it helps in controlling biodegradability and enhancing the mechanical properties by cross-linking and fabrication. Moreover, advanced drug reservoir technology broadens its potential for use in sustained drug release, such as cefazolin and Moxifloxacin biomolecules. The Amnio-M’s content of unique types of stem cells significantly enhances its value as a rich biomaterial for tissue regeneration. In conclusion, advanced technology has significantly enhanced the applications of the Amnio-M in regenerative therapy by both enhancing its forms and delivery methods..

### Future perspectives

The amniotic membrane has many beneficial usages as a natural biocompatible material for tissue engineering applications; many of which have not been thoroughly investigated. It also has some drawbacks, which, if appropriately addressed, can substantially enhance its applications. These drawbacks include rapid degradation, poor mechanical properties, and inconvenient forms. More investigations are thus needed to prepare proper scaffolds forms of Amnio-M in combination with either natural materials, synthetic materials, or hybrids. In addition, the different physicochemical and biomedical properties of these material integrated with the Amnio-M should be thoroughly investigated both in vitro and in vivo to gain insightful information about their interaction with the living cells.

Although the notion of sutureless Amnio-M aimed to decrease the invasiveness of its application in delicate tissue such as the cornea, the use of alternative traditional methods such as glue was not satisfying. Nanotechnology approaches could be superior to conventional glues in providing an adhesive membrane to be directly implanted at the defect site, either in the skin or cornea. By applying new available technologies such as 3D printing, the Amnio-M products are expected to witness significant modifications through integration with different polymers, leading to expanding their clinical applications.

## Data Availability

All data presented in this review are totally available and present in the text.

## Abbreviations

3D: Three-dimensional
ACE: Amnion cytokine extract
AECs: Amniotic epithelial cells
AF: Amniotic fluid
AF-AECs: Human amniotic fluid epithelial cells
AF-CM: Amniotic fluid conditioned media
AFCs: Amniotic fluid stem cells
Al_2_(SO_4_)_3_: Aluminum sulfate
AMEED: Amniotic membrane extract eye drops
Amnio-M: Amniotic membrane
AMSCs: Amniotic mesenchymal stromal cells
BM: Bone marrow
DMEM: Dulbecco's Modified Eagle Medium
DP: Dental pulp
ECM: Extracellular matrix
EMT: Epithelial-mesenchymal transition
ESCs: Embryonic stem cells
FDAM: Freeze-dry Amnio-M
FGF: Fibroblast growth factor
GelMA: Methacrylated gelatin
HA: Hyaluronic acid
IGFBP-4: Insulin-like growth factor binding protein 4
IL-6: Interleukin 6
LSCD: Limbal stem cell deficiency
M-CSFR: Macrophage colony-stimulating factor receptor
MMP: Matrix metalloproteinase
MPO: Neutrophil myeloperoxidase
NT-4: Neurotrophin-4
OA: Osteoarthritis
PDGF: Platelet-derived growth factor
PLCL: Poly-(l-lactide-co-E-caprolactone)
PLGA: Poly-lactic co-glycolic acid
PRP: Platelet-rich plasma
PTX3: Pentraxin 3
SDF-1: Stromal cell-derived factor-1
SJS: Stevens-Johnson syndrome
SNAIL1: Zinc finger protein of Snail1
TEN: Toxic epidermal necrolysis
TGF-β: Transforming growth factor beta
TIMP: Tissue inhibitors of metalloproteases
TNF-α: Tumor necrosis factor alpha
UC-MSCs: Umbilical cord MSCs
UV: Ultraviolet
VEGF: Vascular endothelial growth factor
ZEB: Zinc finger E-box
μ-dHACM: Micronized dehydrated human amnion/chorion membrane
